# Comparison of obecabtagene autoleucel versus an external control arm in adult patients with relapsed/refractory B-cell acute lymphoblastic leukemia

**DOI:** 10.1038/s41375-026-03045-7

**Published:** 2026-07-17

**Authors:** Max S. Topp, Bijal D. Shah, Elias Jabbour, Jae H. Park, Paul Shaughnessy, Aaron C. Logan, Karamjeet S. Sandhu, Mehrdad Abedi, Michael R. Bishop, Daniel J. DeAngelo, Xiang Yin, Ruthanna Davi, Katharine Hodby, Ram Malladi, Wolfram Brugger, Justin Shang, Claire Roddie, Hagop M. Kantarjian

**Affiliations:** 1 University Hospital of Würzburg, Würzburg, Germany; 2 Moffitt Cancer Center, Tampa, FL USA; 3 University of Texas MD Anderson Cancer Center, Houston, TX USA; 4 Memorial Sloan Kettering Cancer Center, New York, NY USA; 5 Methodist Hospital, San Antonio, TX USA; 6 University of California at San Francisco, San Francisco, CA USA; 7 City of Hope National Medical Center, Duarte, CA USA; 8 University of California Davis, Davis, CA USA; 9 University of Chicago, Chicago, IL USA; 10 Dana-Farber Cancer Institute, Boston, MA USA; 11 Medidata, New York, NY USA; 12 University Hospitals Bristol NHS Foundation Trust, Bristol, UK; 13 Cambridge University Hospitals NHS Foundation Trust, Cambridge, UK; 14 Autolus Therapeutics, Munich, Germany; 15 Autolus Therapeutics, Rockville, MD USA; 16 University College London Cancer Institute, London, UK

**Keywords:** Acute lymphocytic leukaemia, Haematological cancer

## Abstract

In the single-arm Phase Ib/II FELIX study (NCT04404660), obecabtagene autoleucel (obe-cel; CD19-directed autologous CAR T-cell therapy) demonstrated high overall remission rates (ORR) and a favorable safety profile in adults with relapsed/refractory (R/R) B-cell acute lymphoblastic leukemia (B-ALL). To contextualize results from FELIX, we compared the efficacy and safety of obe-cel with matched external control arms (ECA) derived from historical trials using propensity score matching. ECAs represented standard of care (SoC) non-CAR T-cell therapies: blinatumomab, inotuzumab ozogamicin, and conventional chemotherapy. The primary endpoint was ORR; secondary endpoints included overall survival (OS). Event-free survival (EFS) and safety were exploratory endpoints. Among the intent-to-treat population in FELIX (*n* = 107), obe-cel demonstrated significantly higher ORR than non-CAR T-cell therapies (67.3% vs 51.4%; odds ratio 1.9; *p* = 0.0257). Median OS was longer with obe-cel when censoring for hematopoietic stem cell transplant (15.1 vs 7.0 months; *p* = 0.0015) and without censoring (13.9 vs 7.8 months; *p* = 0.0430). EFS was significantly improved with obe-cel (median 9.8 vs 2.5 months; *p* < 0.0001). Safety profiles were comparable between groups, with similar rates of Grade ≥3 adverse events. Obe-cel offers superior remission rates and survival benefits over current SoC non-CAR T-cell therapies, with an acceptable safety profile; its use could address unmet needs in adult R/R B-ALL.

## Introduction

B-cell acute lymphoblastic leukemia (B-ALL) presents challenges for clinical management owing to its heterogeneous biology and aggressive nature [1]. Although intensive combination chemotherapy regimens and other immunotherapies have enhanced survival prospects, these therapies are associated with substantial toxicities, while the overall survival rates with these therapies decline as age and the required number of salvage regimens increases [1–4]. Approximately 5–10% of patients with B-ALL will become refractory to primary chemotherapy and 30–60% will relapse [5].

Modern treatment advances including the use of blinatumomab, inotuzumab ozogamicin, and tyrosine kinase inhibitors (TKIs) targeting *BCR-ABL1* kinases (for Philadelphia chromosome [Ph]-positive ALL), have driven rapid evolution in the recommended treatment paradigm for B-ALL [2, 5] and improved patient outcomes [1, 2, 5]. Allogeneic hematopoietic stem cell transplant (HSCT) offers a potentially curative treatment option, but its suitability is limited for many patients due to high rates of post-transplant morbidity and mortality [5–7]. Additionally, most patients are not eligible to proceed to allogeneic HSCT because of the requirement to achieve deep measurable residual disease (MRD)-negative remission to improve post-transplant outcomes; however, achieving such a response in the relapsed/refractory (R/R) setting remains challenging [8]. There is a continuing need for treatments that offer the opportunity to increase the depth and durability of remissions in R/R B-ALL.

In the last decade, CD19-directed chimeric antigen receptor (CAR) T-cell therapies emerged as a promising strategy and received regulatory approval for patients with R/R B-ALL, including tisagenlecleucel (tisa-cel; approved in children and young adults aged ≤25 years) and brexucabtagene autoleucel (brexu-cel; approved in adults aged ≥18 years in the US and ≥26 years in the European Union and UK) [9–14]. Approvals for tisa-cel and brexu-cel were supported by the results of the single-arm Phase I/II ELIANA and ZUMA-3 trials, respectively [7, 15]. High remission rates exceeding 70% were reported in both trials; however, long-term data from the ZUMA-3 trial (median follow-up 41.6 months) showed only 11.5% of patients treated with brexu-cel have ongoing remission without subsequent therapies [7, 15, 16]. Unlike tisa-cel and brexu-cel, which both use the same high-affinity single-chain variable fragment ([scFv]; FMC63) to recognize CD19, obecabtagene autoleucel (obe-cel) is a recent CAR T-cell therapy that uses a different scFv with intermediate affinity due to a fast binding off-rate, as part of its design, to improve persistence and lower toxicity [17, 18]. It has been approved in the R/R B-ALL landscape for adults aged ≥18 years in the US and received conditional marketing authorization for adults aged ≥18 years in the UK and for adults aged ≥26 years in the European Union [18–21]. Obe-cel was associated with durable CAR T-cell persistence and a low incidence of Grade ≥3 immunotoxicities in the Phase Ib/II FELIX trial (NCT04404660) and ALLCAR19 (NCT02935257), an earlier Phase I trial of patients with R/R B-ALL [18, 22]. In the FELIX trial, 40.4% of patients remained in ongoing remission without subsequent HSCT or other therapy, at a median follow-up of 21.5 months [23].

As the range of available treatments expands for patients with R/R B-ALL, questions arise regarding the optimal selection, sequencing, and timing of different therapies [1, 2]. Currently, there is a lack of comparative clinical trial data to determine the relative efficacy and safety of alternative treatment options for R/R B-ALL or inform the choice between CAR T-cell and standard of care (SoC) non-CAR T-cell therapies in this setting. When treatments have not been directly compared in randomized controlled trials (RCTs), it can be appropriate to implement indirect comparisons that utilize an external control arm (ECA) [24]. This is particularly the case in the fields of oncology, hematology, and rare diseases, where patient numbers are limited and/or RCTs are difficult to conduct, at times due to lack of opportune funding or lack of equipoise in the treatment arms [24]. An ECA comprises data from patients outside of the trial using robust statistical methods to provide confidence that their baseline characteristics are well matched to the investigational treatment arm [24] (e.g., ensuring sufficient similarity, homogeneity, and consistency). This allows any differences in outcome between study patients and the ECA to be reliably attributed to the investigational product.

Here we report the findings of a comparison of the efficacy and safety of obe-cel in patients with R/R B-ALL in the FELIX study versus patients treated with SoC in matched ECAs, using a propensity score-based approach. Propensity score matching is an established statistical method that balances the distribution of baseline characteristics and risk factors to reduce potential sources of confounding bias when matched patient sets are compared [25, 26].

## Methods

### Study design and populations

This prospectively designed, non-interventional study used patient-level data from the FELIX trial and global historical trials (HTs). FELIX was an open-label, multi-center, global, single-arm, Phase Ib/II study evaluating the safety and efficacy of obe-cel in patients aged ≥18 years with CD19-positive R/R B-ALL; the study design and primary outcomes have been described previously [18]. Eligible patients underwent leukapheresis to allow manufacture of obe-cel, and bridging therapy, except with blinatumomab, was permitted at the investigator’s discretion [18]. Obe-cel was administered in tumor burden-guided split dosing after lymphodepletion (Day 1), with bone marrow (BM) assessment mandated before lymphodepletion to guide the dose [18]. The second obe-cel dose was given in the absence of severe or unresolved toxic effects on Day 10 (with a permitted window of flexibility of up to two days before or after that date), to a total target dose of 410×10^6^ CAR T-cells. All patients provided written informed consent, and after the institutional review board approved the protocol, the study was conducted in accordance with the principles of the Declaration of Helsinki.

The matched ECA comparison analysis used data from cohort IIA of FELIX, which comprised adult patients with R/R B-ALL and the presence of ≥5% BM blasts at screening [18]. Two populations were analyzed: all patients enrolled in the cohort (intent-to-treat [ITT] population; *n* = 112) and all enrolled patients who were infused with obe-cel (modified ITT [mITT] population; *n* = 94). The target frameworks for the ITT and mITT comparisons are shown in Supplementary Fig. 1.

**Fig. 1:**
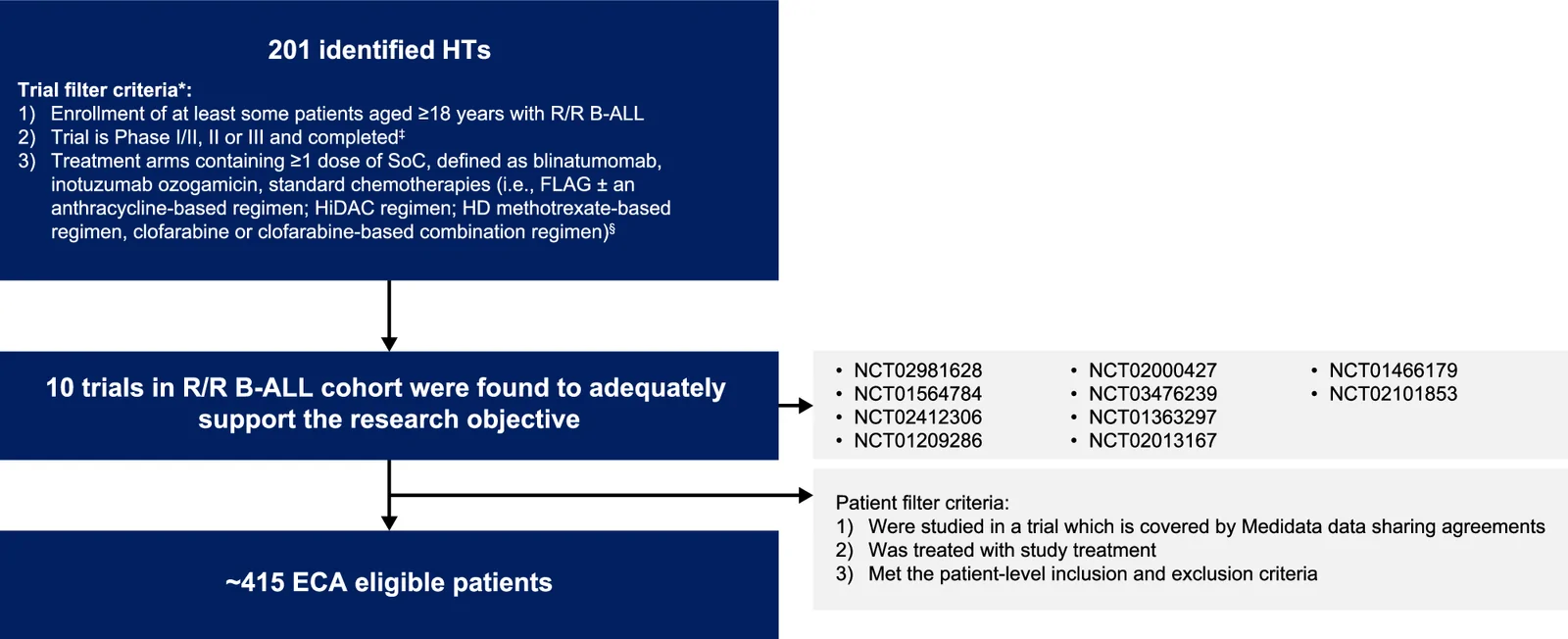
Selection of HTs for the matched ECA comparison analysis. *Sources used include NIH, EudraCT, PubMed, etc.; ^‡^Complete according to the Medidata completion eligibility requirements; ^§^Studies that only enrolled patients with MRD were excluded. ALL acute lymphoblastic leukemia, ECA external control arm, FLAG fludarabine, cytarabine, and filgrastim, HD high-dose, HiDAC high-dose cytarabine, HT historical trial, MRD measurable residual disease, R/R relapsed refractory, SoC standard of care.

The selection process for HTs used in the ECAs is shown in Fig. 1. HTs were retrieved from the Medidata Enterprise Data Store database (which stores patient-level data from thousands of trials, recorded through the Medidata electronic data capture system) and selected by Medidata AI using Data Validation, based on the following criteria: enrollment of at least some patients aged ≥18 years with R/R B-ALL; Phase I/II, II or III and completed; treatment arms contained ≥1 dose of SoC, defined as blinatumomab, inotuzumab ozogamicin, and standard chemotherapies. Of the 201 identified HTs, 10 trials were found to be adequate for supporting the research objectives in terms of study design and excluding studies that only enrolled patients who were in morphologic remission with MRD. The selected trials were open-label, Phase II or Phase III global trials; key features of the studies alongside FELIX are shown in Supplementary Table 1.

Patient filter criteria were applied to the selected HTs to determine eligibility for the ECA, including: being studied in a trial covered by Medidata data sharing agreements and receiving ≥1 dose of study treatment (as defined above); aged ≥18 years with a confirmed diagnosis of R/R B-ALL with ≥5% BM blasts, and Eastern Cooperative Oncology Group (ECOG) performance status of 0 or 1 at start of treatment. Patients with Ph+ B-ALL were required to have TKI contraindicated, be intolerant to TKI, or have failed two lines of TKI therapy (or one line of second-generation TKI). Patients were excluded for isolated extramedullary disease, Burkitt’s lymphoma or chronic myelogenous leukemia; central nervous system pathology or disease; active human immunodeficiency virus, human T-cell lymphotropic virus-1 or 2, hepatitis B or C, or active or uncontrolled fungal, bacterial, viral or other infection requiring systemic antimicrobials; prior allogeneic HSCT within 3 months, or graft versus host disease.

### Propensity score-matching method

Two separate ECAs were created to match with the patients included in FELIX to assess the relative effect of the obe-cel treatment regimen compared with SoC in patients with R/R B-ALL who were enrolled in FELIX (ITT comparison analysis) or had received at least one infusion of obe-cel (mITT comparison analysis). FELIX patients were matched with ECA patients from HTs using a propensity score matching approach. Propensity score was defined as the probability of a patient being enrolled to FELIX (ITT) or infused with obe-cel (mITT), conditional on the key baseline characteristics and prognostic factors, using logistic regression. Baseline characteristics were defined at screening for patients in the ITT analysis and at lymphodepletion for patients in the mITT analysis. Balancing the propensity score between the FELIX investigational arm and the ECA reduces confounding by ensuring that the observed baseline covariates are similar between groups. The matched characteristics and factors between groups included: age at enrollment, sex, ECOG performance status, BM blast percentage at baseline, number of prior lines of therapy, presence of extramedullary disease at baseline, prior allogeneic HSCT, and refractoriness to last prior line of therapy. Matching of patients in FELIX and the ECAs was conducted using the algorithm of greedy nearest-neighbor without replacement and a fixed 1-to-1 matching ratio (which aligns with the commonly used 1:1 randomization ratio in clinical trials). Absolute standardized differences in baseline covariate means between patients in FELIX and the ECAs were computed and compared to ensure balance. BM blast percentage was included as a continuous covariate in the propensity score model, which used values at screening for the ITT analysis and values at lymphodepletion (rather than screening) for the mITT analysis to account for the potential effects of bridging therapy. Further details are provided in the Supplementary Appendix.

### Endpoints

Efficacy (ITT and mITT) and safety endpoints (mITT only) were analyzed and compared between patients from the FELIX investigational arm and ECAs using the post-matching analysis groups. The primary endpoint was overall remission rate (ORR), defined as the proportion of patients achieving complete remission (CR) or CR with incomplete hematologic recovery (CRi). The secondary endpoint was overall survival (OS), defined as the time from either the FELIX enrollment date (ITT), first infusion of obe-cel (mITT), or the start date of SoC administration (ECA), to the date of death due to any reason. In a supplementary analysis, OS was censored on the last contact date, or on the date of allogeneic HSCT if censoring for post-treatment HSCT, whichever was earlier. Exploratory endpoints included event-free survival (EFS), defined as the time from either the FELIX enrollment date (ITT), first infusion of obe-cel (mITT), or start date of SoC administration (ECA), to the earliest of: treatment failure (i.e., not achieving CR/CRi before starting a non-protocol anti-cancer therapy for R/R B-ALL, or death due to underlying disease, or discontinuation from the study except for death due to a reason other than underlying disease), morphological relapse disease, or death due to any cause. EFS was censored on the last adequate assessment date, date of last adequate assessment prior to non-protocol specified therapy, or date of allogeneic HSCT if censoring for post-treatment HSCT, whichever was earlier. Safety was also assessed as a secondary endpoint. Adverse events were coded using the Medical Dictionary for Regulatory Activities (MedDRA) version 26.0 (March 2023) for both FELIX and ECA data.

### Statistical analyses of results

Baseline characteristics, treatment received in the ECAs, and safety data were summarized using descriptive statistics. The primary efficacy analysis was performed by comparing the difference in ORR between FELIX and ECA patients using Fisher’s exact test at a two-sided 5.0% level. In addition, odds ratios together with associated 95% confidence intervals (CI) were estimated for the FELIX patients versus ECAs from a logistic regression model with treatment group (FELIX vs ECA) as a single covariate. Time-to-event endpoints (OS and EFS) for all patient groups were analyzed using the Kaplan-Meier method and compared using a two-sided log-rank test with a 5.0% significance level. Hazard ratios (HR) and the 95% CIs were estimated using a Cox proportional hazard regression model, with the treatment group as a covariate. SAS software (version 9.4 or higher) was used for all statistical analyses.

## Results

### Propensity score matching

The distribution of propensity scores and standardized differences in baseline demographics and disease characteristics between treatment groups before and after matching for the FELIX ITT and mITT groups and the ECAs eligible patients is shown in Supplementary Figs. S2, S3, and S4.

### Patient disposition

A total of 415 patients from ten HTs were found to meet the eligibility criteria of the ECAs (Fig. 1). From the cohort IIA of FELIX, 107 of 112 patients in the ITT group and 84 of 94 patients in the mITT group were matched to ECA1 and ECA2, respectively (see Supplementary Results for details).

### Baseline characteristics and prior treatment

Table 1 summarizes baseline characteristics pre- and post-matching. The characteristics of matched patients were generally well-balanced, with some exceptions. At screening, all patients had ≥5% baseline BM blast across both the FELIX ITT and ECA1 groups; however, a higher proportion of patients had <5% BM blasts at lymphodepletion in the FELIX mITT group (21.4%) compared with patients in ECA2 (0%). In the ITT/mITT groups compared with the ECAs, a higher proportion of patients were Hispanic/Latino (30.8%/32.1% vs 4.7%/4.8%), had Ph-positive disease (22.4%/22.6% vs 4.7%/4.8%), and received prior blinatumomab (35.5%/36.9% vs 1.9%/1.2%) or inotuzumab ozogamicin (31.8%/42.9% vs 0.9%/1.2%), respectively. The proportion of patients receiving prior allogeneic HSCT was similar between the ITT and mITT groups and the ECAs (range: 33.6–37.4%).

**Table 1:** Baseline characteristics of the ITT and mITT groups from FELIX and the ECAs from HTs.

Baseline characteristics*	Pre-matching – ITT analysis		Post-matching – ITT analysis^†^		Pre-matching – mITT analysis		Post-matching – mITT analysis^†^	
	FELIX ITT (*n *= 112)	ECA eligible (*n* = 415)	FELIX ITT (*n* = 107)	ECA1 (*n* = 107)	FELIX mITT (*n* = 94)	ECA eligible (*n* = 415)	FELIX mITT (*n* = 84)	ECA2 (*n* = 84)
**Median age, years** **(Q1–Q3)**	49.0 (33.5–62.5)	36.0 (25.0–54.0)	49.0 (33.0–63.0)	50.0 (31.0–63.0)	50.0 (33.0–62.0)	36.0 (25.0–54.0)	49.5 (33.0–60.5)	49.5 (32.0–61.0)
**Male, n (%)**	60 (53.6)	230 (55.4)	58 (54.2)	53 (49.5)	47 (50.0)	230 (55.4)	43 (51.2)	39 (46.4)
**Hispanic/Latino, n (%)**	33 (29.5)	25 (6.0)	33 (30.8)	5 (4.7)	29 (30.9)	25 (6.0)	27 (32.1)	4 (4.8)
**Baseline ECOG performance status, n (%)**								
0	39 (34.8)	190 (45.8)	37 (34.6)	39 (36.4)	33 (35.1)	190 (45.8)	29 (34.5)	26 (31.0)
1	72 (64.3)	225 (54.2)	69 (64.5)	68 (63.6)	54 (57.4)	225 (54.2)	49 (58.3)	58 (69.0)
2	0	0	0	0	3 (3.2)	0	2 (2.4)	0
Unknown	1 (0.9)	0	1 (0.9)	0	4 (4.3)	0	4 (4.8)	0
**Ph chromosome-positive, n (%)**	26 (23.2)	16 (3.9)	24 (22.4)	5 (4.7)	25 (26.6)	16 (3.9)	19 (22.6)	4 (4.8)
**Median baseline bone marrow blast, % (Q1–Q3)**	55.7 (20.0–85.0)	71.0 (37.5–86.3)	60.0 (20.0–85.0)	59.0 (20.0–82.6)	41.1 (5.0–87.0)	71.0 (37.5–86.3)	50.0 (6.5–89.0)	52.7 (18.5–81.8)
**Baseline bone marrow blast, n (%)**								
<5%	0	0	0	0	23 (24.5)^§^	0	18 (21.4)^§^	0
≥5– ≤75%	71 (63.4)	229 (55.2)	67 (62.6)	70 (65.4)	40 (42.6)	229 (55.2)	36 (42.9)	56 (66.7)
>75%	41 (36.6)	186 (44.8)	40 (37.4)	37 (34.6)	31 (33.0)	186 (44.8)	30 (35.7)	28 (33.3)
**Prior lines of therapy, n (%)**								
1	34 (30.4)	190 (45.8)	34 (31.8)	32 (29.9)	29 (30.9)	190 (45.8)	28 (33.3)	26 (31.0)
2	43 (38.4)	128 (30.8)	41 (38.3)	46 (43.0)	36 (38.3)	128 (30.8)	33 (39.3)	36 (42.9)
>2	35 (31.3)	97 (23.4)	32 (29.9)	29 (27.1)	29 (30.9)	97 (23.4)	23 (27.4)	22 (26.2)
**Prior allogeneic HSCT, n (%)**	43 (38.4)	130 (31.3)	40 (37.4)	36 (33.6)	36 (38.3)	130 (31.3)	29 (34.5)	29 (34.5)
**Prior blinatumomab, n (%)**	41 (36.6)	3 (0.7)	38 (35.5)	2 (1.9)	33 (35.1)	3 (0.7)	31 (36.9)	1 (1.2)
**Prior inotuzumab ozogamicin, n (%)**	37 (33.0)	3 (0.7)	34 (31.8)	1 (0.9)	43 (45.7)	3 (0.7)	36 (42.9)	1 (1.2)

*Baseline was defined at lymphodepletion for FELIX patients in the mITT analysis and at screening for FELIX patients in the ITT analysis.

^†^The clinical variables included in the propensity score models were: age at enrollment, sex, ECOG performance status, bone marrow blast percentage at baseline, number of prior lines of therapy, presence of extramedullary disease at baseline, prior allogeneic HSCT, and refractoriness to last prior line of therapy.

^§^Baseline bone marrow blast at lymphodepletion for some FELIX patients in the mITT analysis was <5% after receiving bridging therapy. All patients enrolled in cohort IIA of FELIX had ≥5% baseline bone marrow blast at screening.

*ECA* external control arm, *ECOG* Eastern Cooperative Oncology Group, *HSCT* hematopoietic stem cell transplant, *HT* historical trial, *ITT* intent-to-treat, *mITT* modified intent-to-treat, *Ph* Philadelphia, *Q* quartile.

### Investigational treatment and HSCT rates

A summary of investigational treatment is shown in Supplementary Table 2. Overall, 86 (80.4%) of ECA1 patients matched to the ITT group of FELIX received blinatumomab, 19 (17.8%) received standard chemotherapy, and two (1.9%) received inotuzumab ozogamicin; 16 (15.0%) of the ITT group and 35 (32.7%) matched ECA1 patients underwent allogeneic HSCT. Among the ECA2 patients matched to the mITT group, 71 (84.5%) received blinatumomab, 12 (14.3%) received standard chemotherapy, and one (1.2%) received inotuzumab ozogamicin; 15 (17.9%) of the mITT group and 27 (32.1%) matched ECA2 patients underwent allogeneic HSCT. All patients in the mITT and ECA analyses received their respective study treatment; however, in the ITT analysis, 10 (9.3%) patients received bridging therapy only, and 8 (7.5%) patients didn’t receive obe-cel or bridging therapy.

### Efficacy endpoints

The median time from obe-cel infusion to data cut-off was 20.3 months for infused patients in the FELIX study, while the median study follow-up was 17.8 and 18.0 months for matched patients in ECA1 and ECA2, respectively. The comparative efficacy analysis showed that the ORRs with obe-cel in the FELIX ITT (67.3%) and mITT (79.8%) groups were significantly higher than for SoC in the matched ECAs (51.4%, OR 1.9 [95% CI 1.1–3.4], *p* = 0.0257; 54.8%, OR 3.3 [95% CI 1.6–6.5], *p* = 0.0009, respectively; Table 2).

**Table 2:** Remission, OS and EFS rates for obe-cel in FELIX and standard of care in the ECAs from HTs.

	ITT (*n* = 107)		mITT (*n* = 84)	
	FELIX	ECA1	FELIX	ECA2
**ORR, %**	67.3	51.4	79.8	54.8
Rate difference, % (95% CI)	15.9 (2.3–28.8)		25.0 (9.4–38.6)	
OR (95% CI)	1.9 (1.1–3.4)		3.3 (1.6–6.5)	
*p* value*	0.0257		0.0009	
**Kaplan-Meier estimated OS rate, % (95% CI)**^†^				
**With censoring for HSCT**				
6 months	73.0 (63.3–80.5)	59.0 (47.8–68.6)	80.1 (69.5–87.3)	58.6 (45.2–69.8)
12 months	58.9 (48.3–68.1)	29.8 (18.2–42.3)	61.9 (49.6–72.1)	29.6 (16.4–44.1)
18 months	43.5 (32.1–54.3)	18.7 (7.9–33.1)	47.1 (33.7–59.4)	25.4 (12.6–40.5)
24 months	43.5 (32.1–54.3)	NE	35.3 (14.9–56.6)	25.4 (12.6–40.5)
**Without censoring for HSCT**				
6 months	72.6 (63.1–80.1)	61.0 (50.9–69.6)	79.8 (69.5–86.9)	63.1 (51.3–72.7)
12 months	55.3 (45.2–64.2)	38.4 (28.8–48.0)	58.3 (47.1–68.0)	40.3 (29.0–51.2)
18 months	40.1 (30.1–49.9)	30.1 (20.8–39.9)	39.9 (28.2–51.4)	33.6 (22.8–44.8)
24 months	36.3 (26.2–46.6)	28.1 (18.8–38.1)	31.9 (16.3–48.7)	26.2 (14.6–39.2)
**Kaplan-Meier estimated EFS rate, % (95% CI)**^†^				
**With censoring for HSCT**				
6 months	59.1 (49.0–67.8)	30.2 (19.9–41.1)	62.6 (50.9–72.3)	33.5 (21.3–46.1)
12 months	42.2 (32.0–52.0)	10.6 (3.2–23.2)	44.9 (33.0–56.2)	16.1 (5.3–32.1)
18 months	30.9 (20.9–41.4)	10.6 (3.2–23.2)	32.1 (19.9–44.9)	NE
24 months	22.4 (11.0–36.4)	NE	24.1 (9.9–41.7)	NE
**Without censoring for HSCT**				
6 months	57.9 (48.0–66.6)	27.9 (19.0–37.5)	60.7 (49.4–70.2)	33.3 (22.5–44.5)
12 months	36.5 (27.4–45.7)	9.5 (3.9–18.3)	39.5 (28.9–49.9)	13.8 (6.3–24.2)
18 months	26.6 (18.1–36.0)	7.6 (2.7–16.0)	28.3 (18.0–39.4)	9.5 (3.4–19.3)
24 months	20.4 (11.0–31.8)	NE	22.6 (11.1–36.6)	NE

^*^p value estimated for the ORR rate difference using Fisher’s Exact test.

^†^Median time from obe-cel infusion to data cut-off was 20.3 months for infused patients in the FELIX study, while median study follow-up was 18.0 and 17.8 months for matched patients in ECA1 and ECA2, respectively.

*CI* confidence interval, *ECA* external control arm, *EFS* event-free survival, *HSCT* hematopoietic stem cell transplant, *HT* historical trial, *ITT* intent-to-treat, *mITT* modified intent-to-treat, *NE* not estimable, *obe-cel* obecabtagene autoleucel, *OR* odds ratio, *ORR* overall remission rate, *OS* overall survival.

**Fig. 2:**
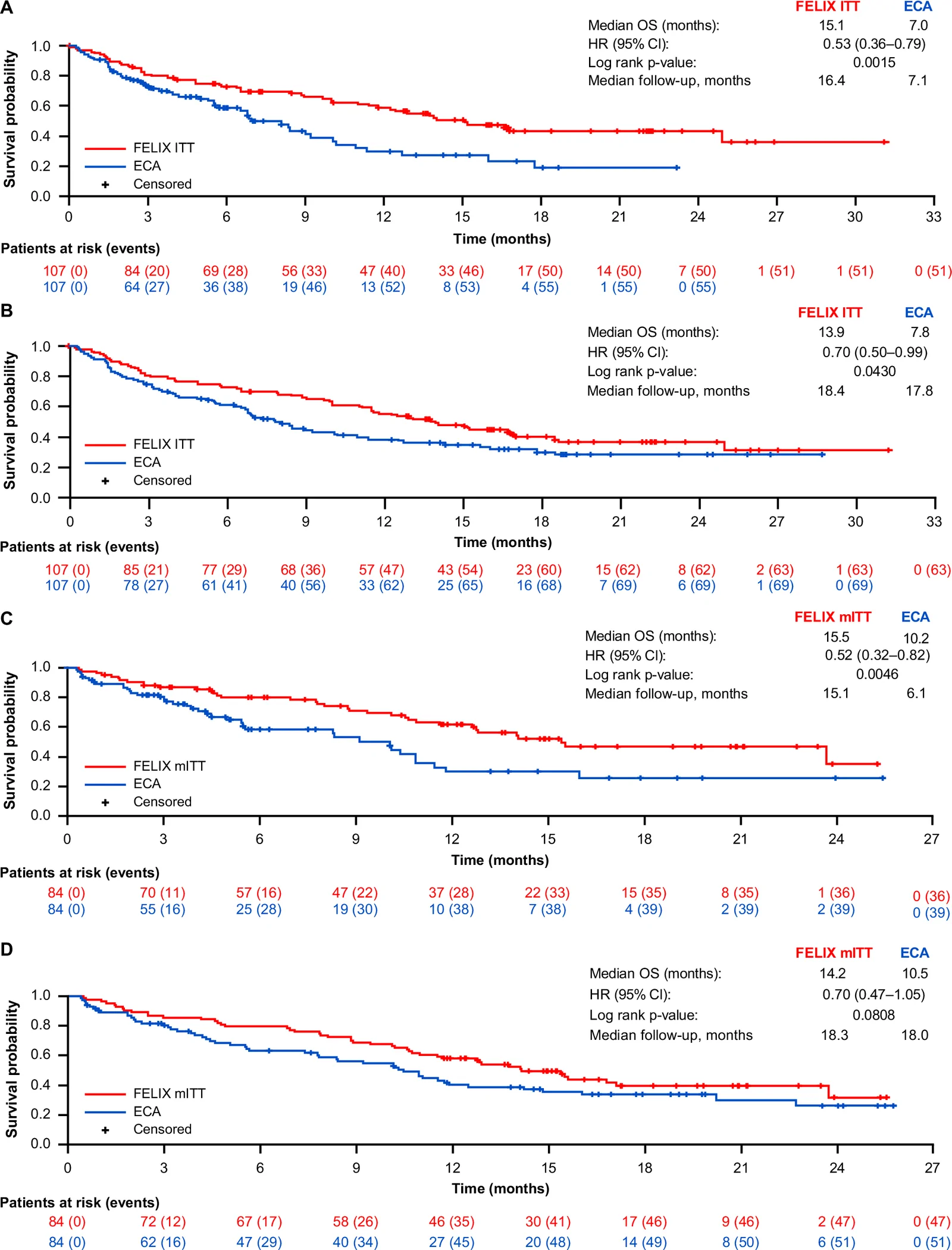
OS with obe-cel in FELIX versus SoC in the ECAs from HTs. **A** ITT with censoring for HSCT. **B** ITT without censoring for HSCT. **C** mITT with censoring for HSCT.* **D** mITT without censoring for HSCT.* *Survival curves estimated using Kaplan-Meier method and compared between groups using two-sided log-rank test with a 5.0% significance level. CI confidence interval, ECA external control arm, HR hazard ratio, HSCT hematopoietic stem cell transplant, HT historical trial, ITT intent-to-treat, mITT modified intent-to-treat, obe-cel obecabtagene autoleucel, OS overall survival, SoC standard of care.

In the ITT analysis with ECA1, the OS was significantly longer with obe-cel than with SoC when censoring for HSCT. The median OS was 15.1 months in the ITT group versus 7.0 months in ECA (HR 0.53 [95% CI 0.36–0.79], *p* = 0.0015; Fig. 2A). Without HSCT censoring, a trend for longer OS with obe-cel versus SoC was observed: median OS 13.9 months in the ITT group versus 7.8 months in ECA (HR 0.70 [95% CI 0.50–0.99], *p* = 0.0430; Fig. 2B). Kaplan-Meier estimated OS rates with obe-cel were higher in the ITT group with or without censoring for HSCT than with SoC in the ECAs (Table 2). At 18 months, the OS rates with censoring for HSCT were 43.5% in the ITT group versus 18.7% in ECA; without censoring for HSCT, they were 40.1% in the ITT group versus 30.1% in ECA.

These results for OS were consistent in the mITT analysis with ECA2; the median OS when censoring for HSCT was 15.5 months in the mITT group compared with 10.2 months in ECA (HR 0.52 [95% CI 0.32–0.82], *p* = 0.0046; Fig. 2C). The median OS without HSCT censoring was 14.2 months with obe-cel in the mITT group compared with 10.5 months in ECA (HR 0.70 95% CI 0.47–1.05, *p* = 0.0808; Fig. 2D). Kaplan-Meier estimated OS rates with obe-cel were higher in the mITT group with or without censoring for HSCT than with SoC in the ECA. At 18 months, the OS rates with or without censoring for HSCT were 47.1%/39.9% in the mITT group compared with 25.4%/33.6% for SoC in ECA.

**Fig. 3:**
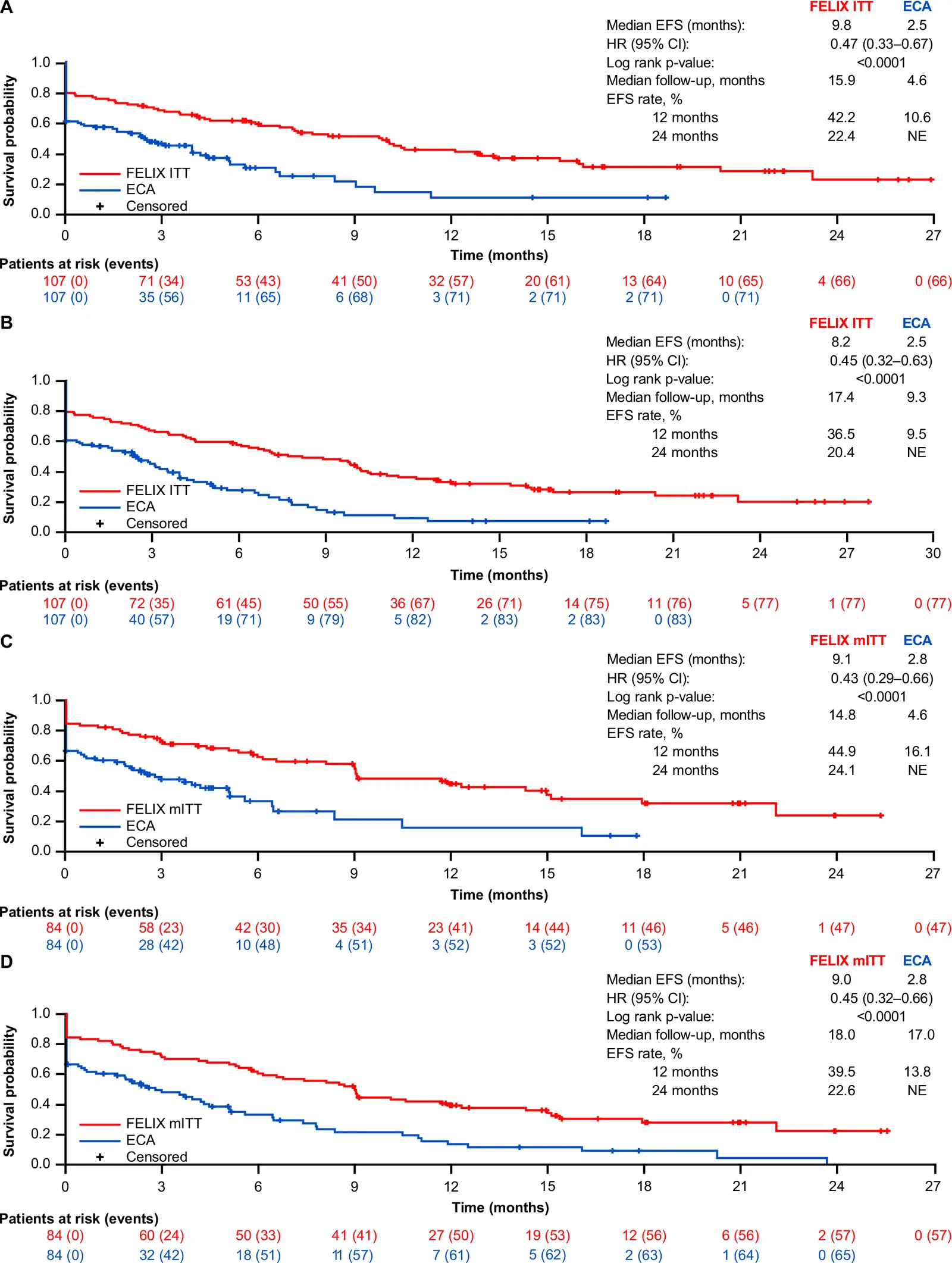
EFS with obe-cel in FELIX versus SoC in the ECAs from HTs. **A** ITT with censoring for HSCT. **B** ITT without censoring for HSCT. **C** mITT with censoring for HSCT.* **D** mITT without censoring for HSCT.* *Survival curves estimated using Kaplan-Meier method and compared between groups using two-sided log-rank test with a 5.0% significance level. CI confidence interval, ECA external control arm, EFS event-free survival, HR hazard ratio, HT historical trial, HSCT hematopoietic stem cell transplant, ITT intent-to-treat, mITT modified intent-to-treat, NE not estimable, obe-cel obecabtagene autoleucel, SoC standard of care.

In the ITT analysis with ECA1, EFS with censoring for HSCT was significantly longer with obe-cel versus SoC. Median EFS was 9.8 months in the ITT group compared with 2.5 months in ECA (HR 0.47 [95% CI 0.33–0.67], *p* < 0.0001; Fig. 3A). Similar results were obtained without censoring for HSCT: median EFS 8.2 months versus 2.5 months in the ITT group and ECA, respectively (HR 0.45 [95% CI 0.32–0.63], *p* < 0.0001; Fig. 3B). Kaplan-Meier estimated EFS rates with obe-cel were higher in the ITT group with or without censoring for HSCT when compared with SoC in the ECA (Table 2).

These results for EFS were consistent in the mITT analysis with ECA2; median EFS when censoring for HSCT was 9.1 months in the mITT group compared with 2.8 months in ECA (HR 0.43 [95% CI 0.29–0.66], *p* < 0.0001; Fig. 3C). The median EFS without censoring was 9.0 months in the mITT group versus 2.8 months in ECA (HR 0.45 [95% CI 0.32–0.66], *p* < 0.0001; Fig. 3D). Kaplan-Meier estimated EFS rates with obe-cel were higher in the mITT group with or without censoring for HSCT when compared with SoC in the ECA.

Differential (arm-specific) censoring for survival outcomes were conducted in a post-hoc analysis, with obe-cel in the FELIX ITT censored for HSCT and SoC in ECA1 not censored for HSCT. Kaplan-Meier estimated OS and EFS rates were higher with obe-cel compared with ECA1 (Supplementary Fig. S5).

### Safety

Almost all patients experienced a treatment-emergent adverse event (100% with obe-cel in the FELIX mITT group and 97.6% in the ECA); the incidence of Grade ≥3 treatment-emergent adverse events was similar between the mITT and ECA2 (81.0% vs 86.9%) (Table 3). The serious adverse event rate was numerically higher for the mITT group than ECA2, both for any-grade (63.1% vs 44.0%) and Grade ≥3 (51.2% vs 40.5%) events. Obe-cel and SoC had comparable incidence of Grade ≥3 cytokine release syndrome (3.6% vs 3.6%) within 3 months of treatment initiation. Rates of Grade ≥3 adverse events within the system organ classes of nervous system disorders or psychiatric disorders within 3 months of starting treatment were 10.7% with obe-cel and 9.5% with SoC.

**Table 3:** Safety outcomes for obe-cel in the FELIX mITT and standard of care in the ECA from HTs.

TEAE, *n* (%)*	FELIX mITT (*n* = 84)		ECA (*n* = 84)	
	Any grade	Grade ≥3	Any grade	Grade ≥3
Any TEAE	84 (100)	68 (81.0)	82 (97.6)	73 (86.9)
Cytokine release syndrome^†^	64 (76.2)	3 (3.6)	22 (26.2)	3 (3.6)
Infections^†,‡^	43 (51.2)	22 (26.2)	50 (59.5)	24 (28.6)
Nervous system or psychiatric disorders^†,§^	50 (59.5)	9 (10.7)	37 (44.0)	8 (9.5)

^*^Defined as any AE with post-treatment onset. ^†^Within 3-months of treatment initiation. ^‡^FELIX was conducted during the COVID-19 pandemic; six patients with COVID-19 were included in this analysis. ^§^Analysis of nervous system or psychiatric disorders performed using combined MedDRA system organ classes.

*AE* adverse event, *COVID-19* Coronavirus disease 2019, *ECA* external control arm, *HT* historical trial, *MedDRA* Medical Dictionary for Regulatory Activities, *mITT* modified intent-to-treat, *obe-cel* obecabtagene autoleucel, *TEAE* treatment-emergent adverse event.

The proportion of patients with infections within 3 months of any grade was comparable at 56.0% for obe-cel in the mITT group versus 59.5% in ECA2 from HTs; however, this did include six patients in the FELIX study with COVID-19. FELIX was initiated from 2020 (during the COVID-19 pandemic), while the HTs took place between 2014 and 2017 (before the COVID-19 pandemic). When COVID-19 infections were excluded, 51.2% of patients in the mITT group had infections within 3 months of treatment start date, compared with 59.5% of patients in ECA2 (Grade ≥3, 26.2% vs 28.6%, including events caused by an unspecified pathogen [19.0% vs 20.2%], bacterial infection [9.5% vs 8.3%], fungal infection 3.6% vs 3.6%), or virus [0% vs 1.2%].

There were fewer early deaths (within 3 months of treatment initiation) with obe-cel in the mITT group compared with SoC in ECA2 (14.3% vs 19.0%), and a lower proportion of patients with Grade 5 events overall (42.9% vs 60.7%, respectively).

## Discussion

This study used a propensity-based scoring approach to contextualize and compare the efficacy and safety of obe-cel in patients with R/R B-ALL from the FELIX study with patients treated with SoC non-CAR T-cell therapies in ECAs derived from HTs.

The efficacy results in the ITT group in FELIX showed consistently improved outcomes over matched patients assigned to SoC therapy in the ECAs. The primary efficacy endpoint showed a significant 15.9% percentage point increase in ORR with obe-cel versus SoC. With median survival follow-up exceeding 15 months in FELIX, the secondary endpoint analysis showed that estimated median OS was longer in patients receiving obe-cel in the ITT or mITT groups than patients in the ECAs, reaching statistical significance when censoring for HSCT. More patients received HSCT post-treatment in the ECAs than in the ITT or mITT of FELIX, which may have impacted the OS results. A trend for OS benefit with obe-cel compared with the ECAs was observed without censoring for HSCT. Supportive exploratory analyses showed a statistically significant increase in median EFS for patients treated with obe-cel in both the ITT and mITT groups compared with those receiving SoC in the matched ECAs, with and without censoring for post-treatment HSCT. The improvement in outcomes in the ITT population was observed despite 16.8% of patients not receiving obe-cel.

No safety concerns arose from the comparative analysis of obe-cel patients from the FELIX mITT and the matched ECA, with similar rates of treatment-emergent adverse events; no increased risk of infections, nervous or psychiatric disorders, or Grade ≥3 cytokine release syndrome were observed with obe-cel. Despite CAR T-cell therapies being associated with immunological toxicities [7, 15], the safety profile of obe-cel is similar to that of SoC non-CAR T-cell therapies.

These results suggest that obe-cel can offer clinical benefits over SoC non-CAR T-cell therapies for patients with R/R B-ALL, who often have a poor prognosis with limited remaining options [1, 2].

ECAs have been implemented for other single-arm clinical trials investigating CAR T-cell therapies in patients with R/R B-ALL. Tisa-cel was associated with a statistically significant higher likelihood of achieving CR and lower hazard of death in an indirect-treatment comparison with blinatumomab [27] and demonstrated significantly longer OS compared with additional SoC using a matching-adjusted indirect comparison (MAIC) [28]. Results from an MAIC showed improved survival outcomes with brexu-cel compared with blinatumomab and inotuzumab ozogamicin [29]. Additionally, the SCHOLAR-3 trial contextualized the results found in the ZUMA-3 trial by using synthetic control arms derived from individual patient-level data sampled from HTs [30]. The complete response rate at Week 24 was higher in patients treated with brexu-cel versus propensity matched controls and this benefit was maintained at the 2-year follow-up; however, the data was limited by a small data set [30, 31]. Safety outcomes were not compared in the ECA analyses with tisa-cel and brexu-cel [27–31].

The current analysis has limitations. Differences in populations and methodologies, along with evolving clinical practices can produce misleading results; therefore, caution is warranted when interpreting results. Reliance on historical or external data sources can introduce selection bias and confounding [24], and known prognostic factors such as ethnicity or Ph chromosome status were not included due to lack of data feasibility. Moreover, the limited number of matched patients with Ph+ B-ALL in the ECA cohorts compared with the FELIX cohorts may have impacted survival outcomes due to the increased likelihood of TKI utilization. Additionally, survival outcomes attributable to the investigated therapy are frequently confounded by subsequent treatments in this population. In the FELIX trial, 40.4% of patients maintained ongoing remission without receiving subsequent HSCT or other therapies [23]. However, among patients who did undergo HSCT or additional treatments while in remission, it remains challenging to differentiate the confounding effects of these interventions on their clinical outcomes. Comparisons between obe-cel and individual treatments in the ECAs could not be conducted due to agreements governing Medidata’s data collaboration program. The most predominantly used treatment in the ECA was blinatumomab. The number of patients treated with inotuzumab ozogamicin was limited in the ECA cohorts as few eligible patients receiving inotuzumab ozogamicin were identified in the Medidata Enterprise Data Store database. As such, interpretation of results against inotuzumab ozogamicin and conventional chemotherapy as SoC comparators was limited. Furthermore, recent changes in clinical practice regarding blinatumomab, which is now used less frequently in the R/R setting [32], may impact the relevance of survival comparisons found in this study. The same limitation is observed in the SCHOLAR-3 trial where the most predominantly used treatment in the ECA was chemotherapy [31]. Another potential limitation is that approximately 20% of patients in the mITT group had a BM blast <5% after lymphodepletion, which could have confounded the survival outcomes when compared with patients in the ECA2 arm who all had a BM blast ≥5%. However, it is important to note that at screening patients across both FELIX ITT and ECA1 groups had ≥5% BM blasts.

Our analysis was strengthened by use of the propensity-based scoring approach, which is well-established and deemed appropriate where RCTs cannot be conducted and directly comparative data are limited by the size of the available patient pool [24]. The propensity scores allowed prognostic factors to be well-balanced between groups, mitigating potential confounding effects that can arise when comparing outcomes between treatments in the absence of a randomized clinical trial. This offers an advantage over traditional external controls commonly used for single-arm trials, such as benchmarks with medical literature or clinical intuition, where baseline balance may not be present or even known. The outcome analysis was kept blinded until after the composition of the ECAs was shown to be well-balanced with the FELIX study for all baseline characteristics examined. Use of the ECAs from several HTs also allowed detailed quantitative analysis of the treatment effects of obe-cel relative to multiple SoC therapies, which would otherwise not be possible from a single arm trial design. Additionally, the data set used in this study was larger than other similar studies, such as the SCHOLAR-3 trial [30, 31].

In conclusion, treatment with obe-cel in FELIX demonstrated durable efficacy with a higher ORR, and longer OS and EFS versus SoC non-CAR T-cell therapies in matched ECAs from HTs. The safety profile of obe-cel was also favorable with minimal immunological toxicities, being comparable to that of SoC non-CAR T-cell therapies. Results from this propensity matched analysis indicate that obe-cel may improve survival outcomes compared with historical SoC therapies and its use may help to bridge the unmet needs in adult R/R B-ALL.

## Supplementary information

Supplementary Appendix - Clean

## Data Availability

The data that support the findings of this study will be made available to qualified researchers for agreed pre-specified purposes upon written request. All data access requests should be sent to: clinicaltrials@autolus.com.
