# Supplementary material for: Comparison of obecabtagene autoleucel versus an external control arm in adult patients with relapsed/refractory B-cell acute lymphoblastic leukemia
Source: Leukemia. 2026 Jul 17;40(10):2181–90. doi: 10.1038/s41375-026-03045-7 (PMC13612215; doi:10.1038/s41375-026-03045-7)
Supplement: Supplementary file 1 [file 41375_2026_3045_MOESM1_ESM.pdf]

SUPPLEMENTARY APPENDIX

Supplementary Methods

Propensity score estimates and matching algorithm

Propensity score was defined as the probability of a patient enrolling in FELIX (intent-to-treat group) or getting infused to obe-cel (modified intent-to-treat group), conditional on the baseline characteristics and prognostic factors (i.e., potential confounders) using logistic regression:

$$p(x) = P(T = 1|\mathrm{X} = \mathrm{x})$$

where T = 1 if a patient enrolls or gets infused or 0 otherwise; and *X* is a vector representing the covariates included in the propensity score model.

The greedy nearest-neighbor matching algorithm was utilized as follows. After a propensity score was estimated for each patient included in the analysis from both the investigational arm of FELIX and the historical trial (HT) pool, patients from FELIX were randomly ordered, starting from the first patient in the obe-cel arm, matched to an HT patient whose propensity score was closest to that of the FELIX patient and within a prespecified maximum distance (i.e., caliper). A caliper width equal to 0.25 of the pooled standard deviation of logit of the propensity score from the two groups was used [1]. Matching was conducted without replacement (i.e., the matched HT patients were removed from consideration for further matching and the next FELIX patient was then selected). This process was repeated sequentially for all patients in FELIX. The matched patients from the HTs were the components of the external control arms (ECAs).

Post-matching evaluation of covariate balance

The propensity score was used as a balancing score, to examine whether the distribution of measured baseline covariates was similar between patients in FELIX and the ECAs. Absolute standardized differences in covariate means were computed and compared. For a continuous covariate, the standardized difference was calculated as:

$$d = \frac{\bar{x}_t - \overline{x_c}}{\sqrt{(s_t^2 + s_c^2)/2}}$$

Where $\bar{x}_t$ and $\overline{x_c}$ denote the sample mean of the covariate for patients from FELIX and the ECAs, and s_t^2 and s_c^2 denote the sample variance of the covariate, respectively. The sample means and sample variances in formulas are unweighted estimates.

For dichotomous (or categorical) variables, the standardized difference was defined as:

$$d = \frac{\hat{p}_t - \hat{p}_c}{\sqrt{\{\hat{p}_t(1-\hat{p}_t) + \hat{p}_c(1-\hat{p}_c)\}/2}}$$

where $\hat{p}_t$ and $\hat{p}_c$denote the prevalence of covariate (or a category of covariate) for the patients in FELIX and the HTs, respectively. For covariates with more than two categories, the standardized difference for each level of the categorical variable was calculated. If the matching ratio between investigational arm and ECA is not 1:1, (e.g., when full matching is used) weighted absolute standardized differences were calculated to evaluate the balance between FELIX patients and the ECAs. Each sample estimate was replaced by its weighted equivalent. The weighted mean was defined as:

$$\bar{x}_{WT} = \frac{\sum w_i x_i}{\sum w_i}$$

and weighted sample variance was defined as:

$$s_{WT}^2 = \frac{\sum w_i}{(\sum w_i)^2 - \sum {w_i}^2} - \sum w_i \ (x_i - \bar{x}_{WT})^2$$

where w_i is the weight assigned to the ith patient.

The absolute standardized differences should generally be less than 0.25 [2]. An absolute standardized difference of less than 0.10 indicates a negligible difference in the mean or prevalence of a covariate between treatment groups [3].

Supplementary Figure 1. Target trial framework for the A) ITT and B) mITT comparison.

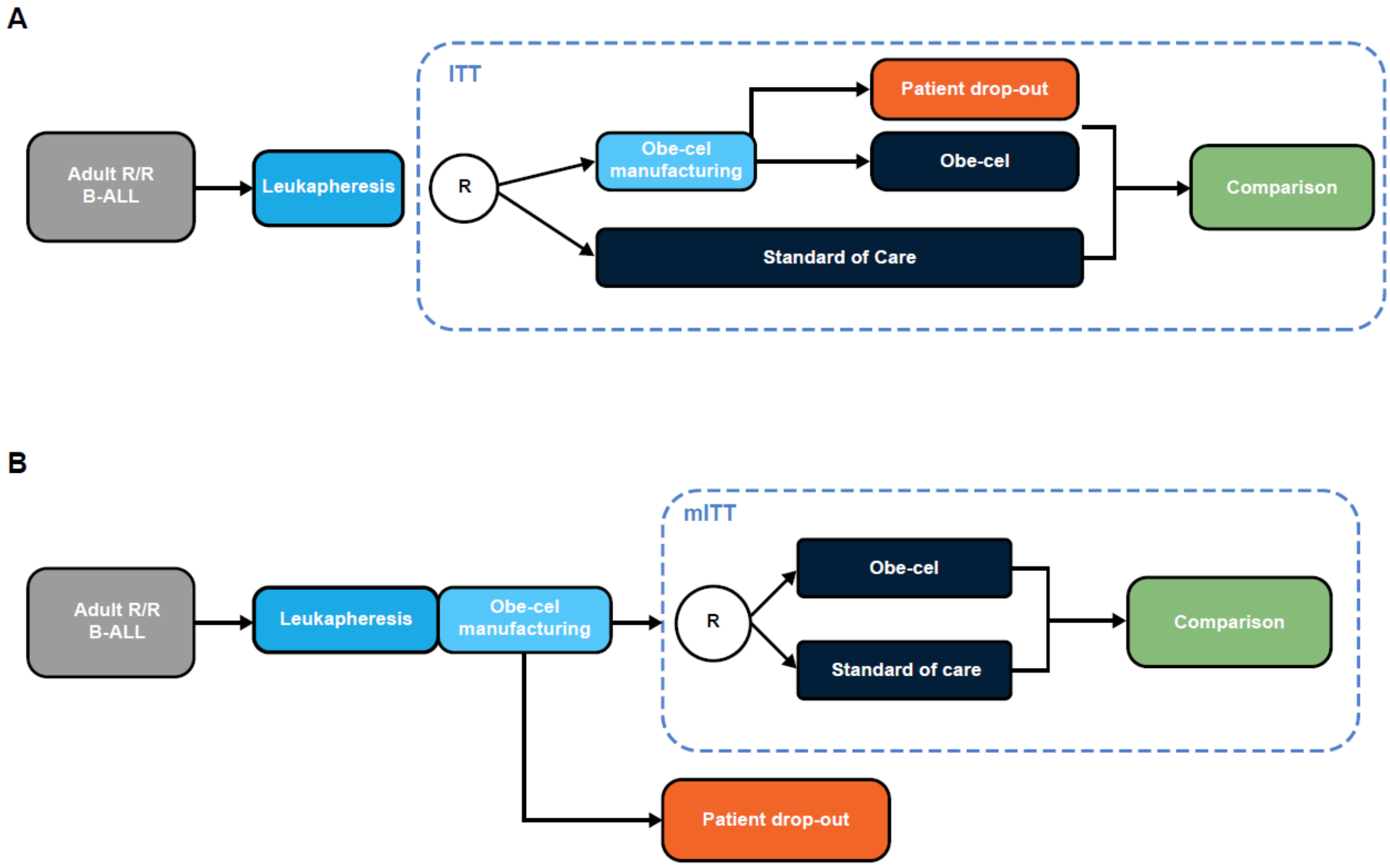

ALL, acute lymphoblastic leukemia; obe-cel, obecabtagene autoleucel; R, randomization; R/R, relapsed/refractory.

Supplementary Results

Supplementary Figure 2 shows the distribution of propensity scores for the FELIX ITT and mITT groups and the eligible patients in the ECAs, and the degree to which these distributions overlap. Each circle represents a patient in the analysis set. In the ITT group, an overlap in the distribution of propensity scores with ECA eligible patients was evident. Only one patient fell outside of the support region and four patients within this region were not matched with any external controls; therefore, five patients were excluded from the ITT comparative analysis. Similarly, overlap in the distribution of propensity scores for the FELIX mITT group and ECA eligible patients was evident, and no patients fell outside of the support region. Ten patients in the mITT group and within the support region were not matched with any external controls, as illustrated with red circles. These patients were excluded from the mITT versus ECA comparison.

Supplementary Figure 2. Distribution of propensity score by treatment group (before/after matching). (A) FELIX ITT group versus ECA1 from HTs (B) FELIX mITT group versus ECA2 from HTs.

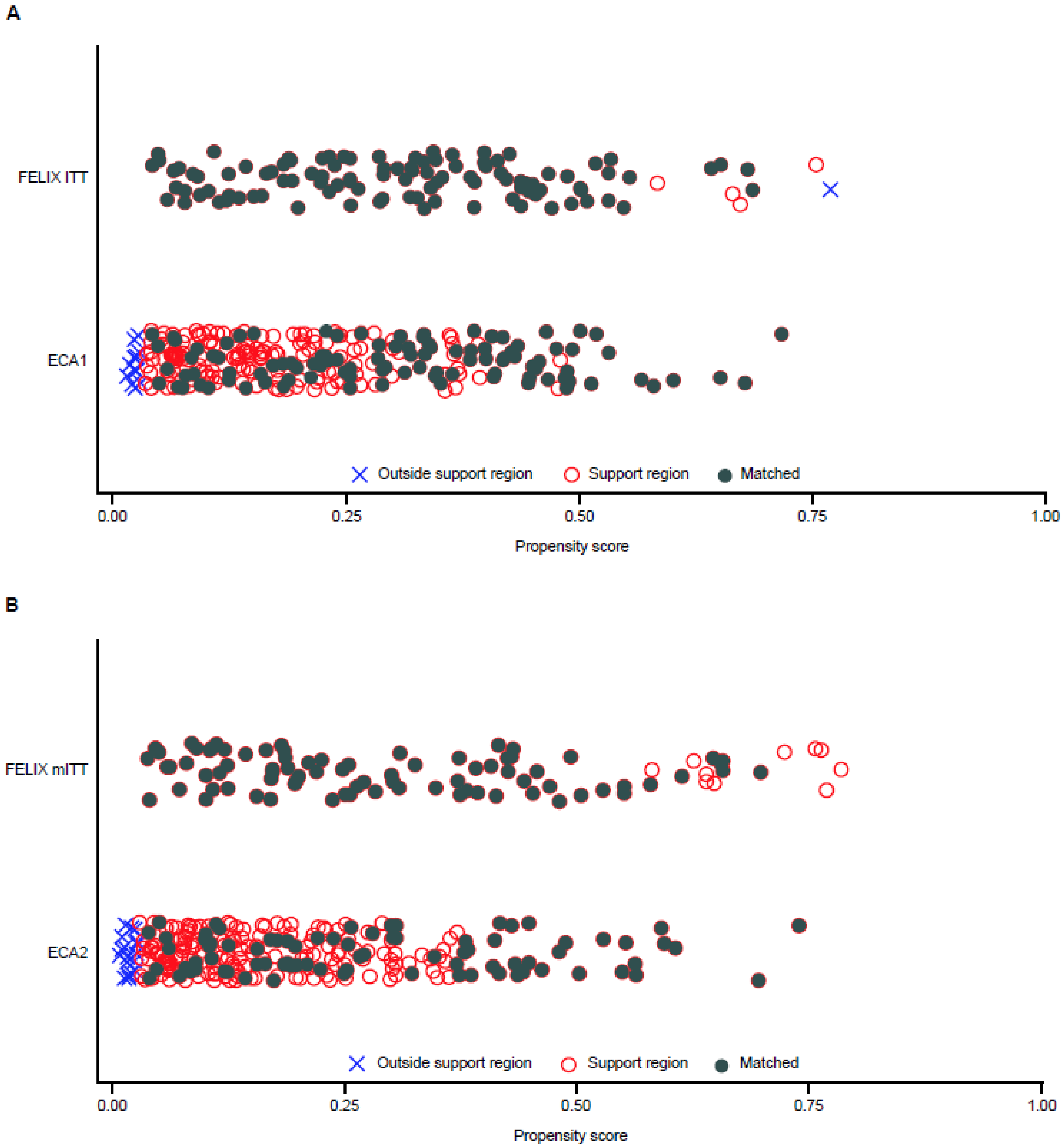

ECA, external control arm; HT, historical trial; ITT, intent-to-treat; mITT, modified intent-to-treat.

Before the ECA could be used for any between-group comparison, the degree of balance was examined for the propensity score as well as the observed individual baseline characteristics that were included in building the propensity score model. **Supplementary Fig. 3** presents box plots illustrating the distributions of the propensity scores for the patients that were included in the before matching set (top) and after matching set (bottom) for the ITT and mITT groups versus the ECAs. After matching, comparability was gained and the median and variability of the groups became similar.

Supplementary Figure 3. Plot of distribution of propensity scores (before/after matching).
(A) FELIX ITT group versus ECA1 from HTs (B) FELIX mITT group versus ECA2 from HTs.

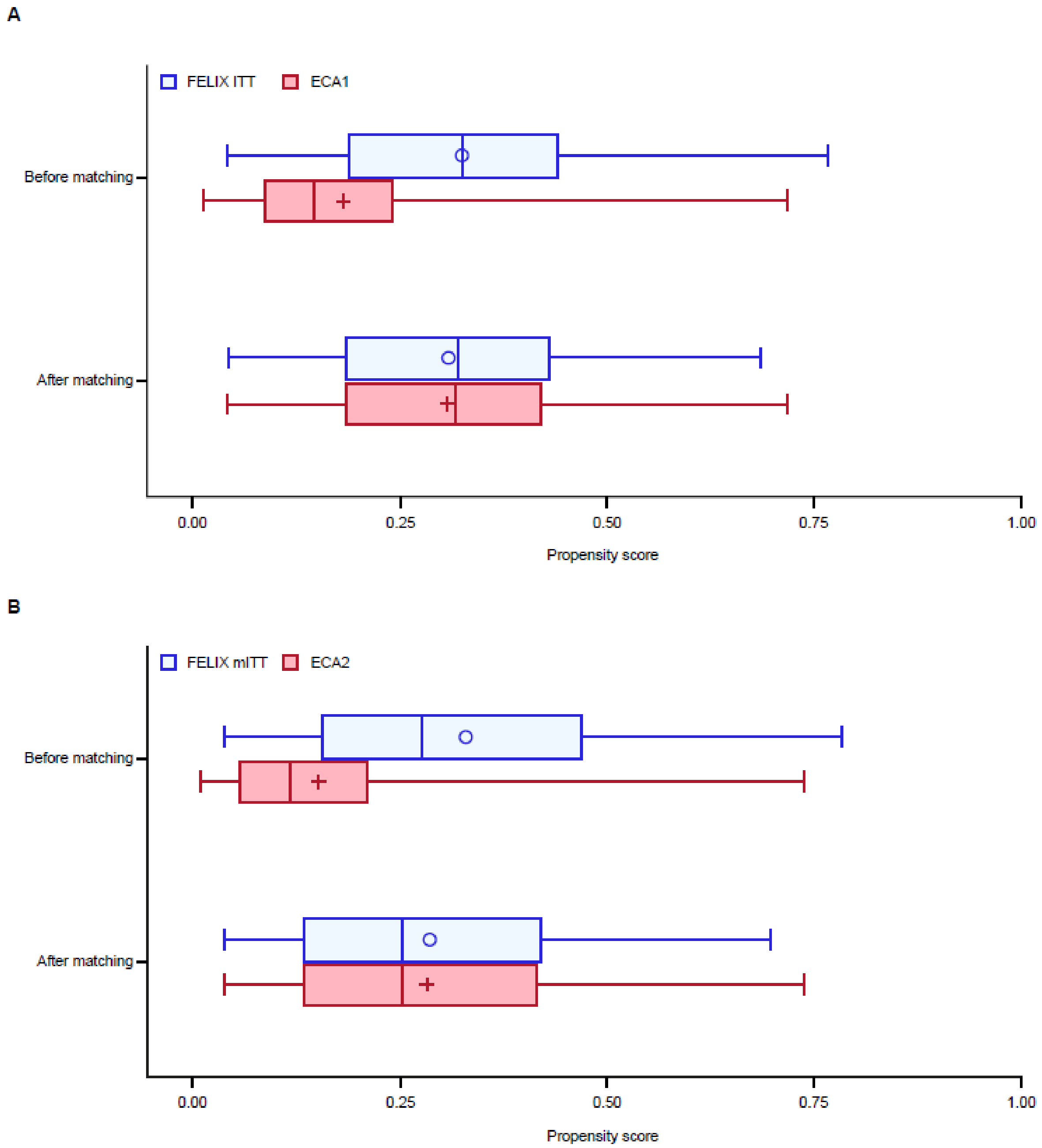

ECA, external control arm; HT, historical trial; ITT, intent-to-treat; mITT, modified intent-to-treat.

Assessment of balance in terms of individual baseline covariates yielded observations consistent with the conclusion of well-balanced groups. **Supplementary Fig. 4** shows the standardized difference between the FELIX ITT and mITT and ECA before and after matching, for each important baseline characteristic. Reductions in the absolute standardized difference between groups for each variable were observed and the absolute standardized differences after matching were well below 0.25 the common threshold for acceptable difference, and all were also below 0.10, the prespecified threshold for designating a negligible difference in the mean or prevalence of a covariate between groups (as described in **Supplementary Methods**, above).

Supplementary Figure 4. Standardized differences in baseline demographics and disease characteristics between treatment groups (before/after matching). (A) FELIX ITT group versus ECA1 from HTs (B) FELIX mITT group versus ECA2 from HTs.

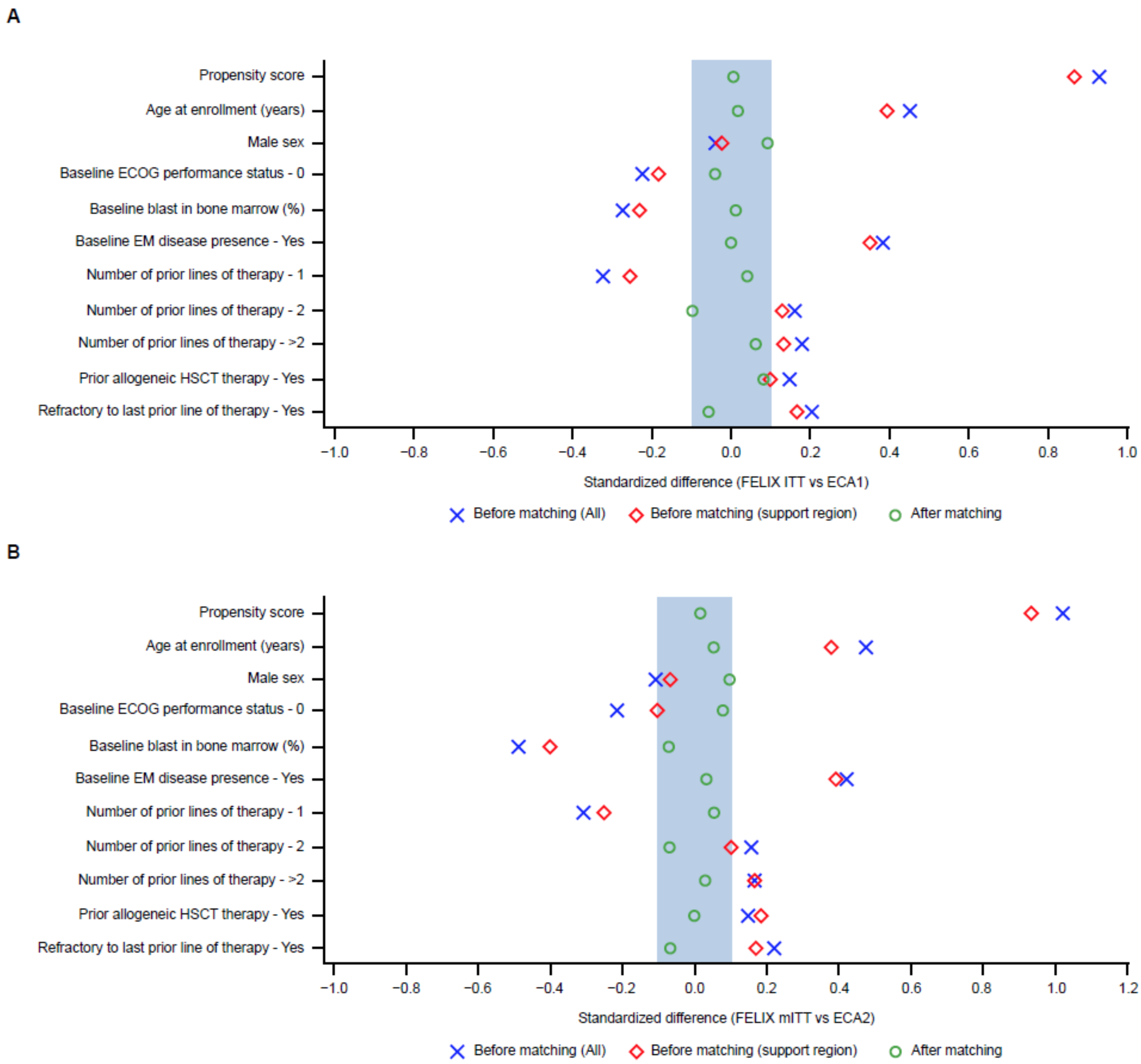

ECA, external control arm; ECOG, Eastern Cooperative Oncology Group; EM, extramedullary; HT, historical trial; HSCT, hematopoietic stem cell transplant; ITT, intent-to-treat; mITT, modified intent-to-treat.

Supplementary Figure 5. Survival outcomes with obe-cel in FELIX ITT (with censoring for HSCT) versus SoC in ECA1 (without censoring for HSCT) from HTs.* (A) Overall survival (B) Event-free survival.

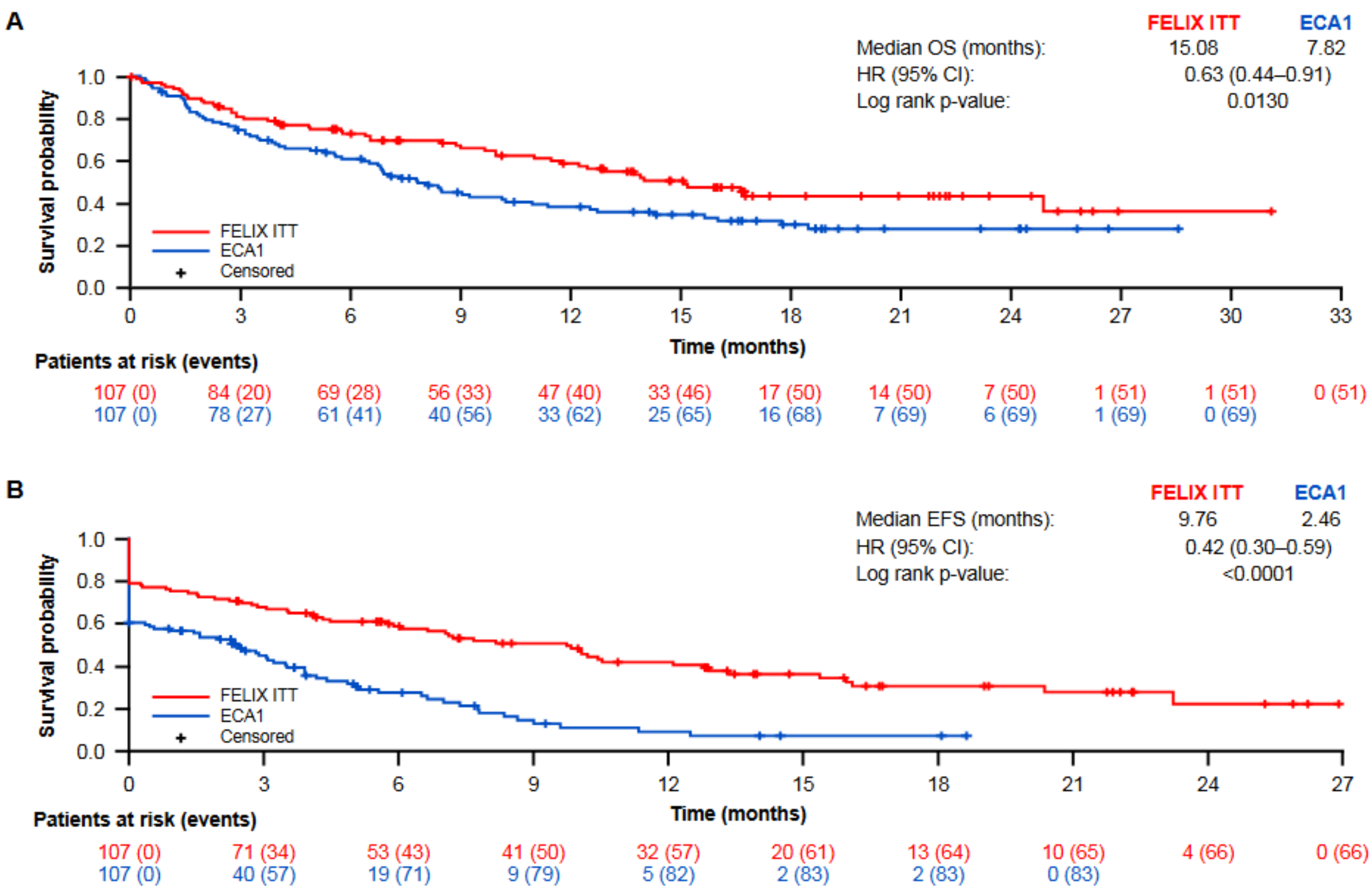

*Survival curves estimated using Kaplan-Meier method and compared between groups using two-sided log-rank test.

CI, confidence interval; ECA, external control arm; EFS, event-free survival; HSCT, hematopoietic stem cell transplant; HT, historical trial; HR, hazard ratio; ITT, intent-to-treat; obe-cel, obecabtagene autoleucel; OS, overall survival; SoC, standard of care.

Supplementary Table 1. Key features of FELIX and the HTs selected for the ECA.

**Features**	**FELIX**	**HTs (from multiple trials)**
Trial design	Open-label, Phase Ib/II	Open-label, Phase II and Phase III
Intervention assignment	Single arm	Single-arm and randomized parallel assignment
Country/region	Spain, United Kingdom and United States	Global
Year of initiation	2020	2014–2017
Study treatment*	Obe-cel treatment regimen including optional bridging, preconditioning therapies, and obe-cel infusion	Blinatumomab<br>Inotuzumab<br>Standard chemotherapies including HiDAC, FLAG regimen, mitoxantrone, methotrexate, or clofarabine or clofarabine-based combination regimens
Main efficacy endpoints	ORR (CR/CRi), CR, DoR, EFS, OS	ORR, CR, CRh, CRi, OS, DoR, EFS

*Study treatments received, by group, are shown in **Supplementary Table 2**.

CR, complete remission; CRh, complete remission with partial hematologic recovery; CRi, complete remission with incomplete hematologic recovery; DoR, duration of remission; ECA, external control arm; EFS, event-free survival; FLAG, fludarabine, cytarabine and filgrastim; HT, historical trial; HiDAC, high-dose cytarabine; obe-cel, obecabtagene autoleucel; ORR, overall remission rate; OS, overall survival.

Supplementary Table 2. Treatment summary by treatment group, after matching.

Treatment, n (%)	ITT (n=107)		mITT (n=84)	
	FELIX	ECA1	FELIX	ECA2
obe-cel	89 (83.2)	0	84 (100.0)	0
Blinatumomab	0	86 (80.4)	0	71 (84.5)
Inotuzumab ozogamicin	0	2 (1.9)	0	1 (1.2)
Standard chemotherapy	0	19 (17.8)	0	12 (14.3)
Bridging therapies only	10 (9.3)	0	0	0
Not received study treatment or bridging therapy	8 (7.5)	0	0	0

ECA, external control arm; HT, historical trial; ITT, intent-to-treat; mITT, modified intent-to-treat; obe-cel, obecabtagene autoleucel.

References

1. Rosenbaum PR, Rubin DB. Constructing a Control Group Using Multivariate Matched Sampling Methods That Incorporate the Propensity Score. Am Stat. 1985; 39(1):33–8.
2. Osborne J, Stuart EA, Rubin DB. Best Practices in Quasi–Experimental Designs: Matching Methods for Causal Inference. In: Osborne J (ed). Best Practices in Quantitative Methods. SAGE Publications, Inc, 2008. pp 155–176.
3. Normand ST, Landrum MB, Guadagnoli E, Ayanian JZ, Ryan TJ, Cleary PD, et al. Validating recommendations for coronary angiography following acute myocardial infarction in the elderly: a matched analysis using propensity scores. J Clin Epidemiol. 2001; 54(4):387–98.
